# A novel *SBF1* missense mutation causes autosomal dominant Charcot–Marie–Tooth disease type 4B3

**DOI:** 10.3389/fneur.2024.1495711

**Published:** 2024-11-22

**Authors:** Huaqi Liu, Jing Dong, Zhe Xie, Li Yu

**Affiliations:** ^1^Department of Trauma and Microsurgery Orthopedics, Zhongnan Hospital of Wuhan University, Wuhan, China; ^2^Clinical Research Center for Microsurgical Orthopedics of Hubei Province, Zhongnan Hospital of Wuhan University, Wuhan, China

**Keywords:** CMT4B3, SBF1, MTMR5, autosomal dominant, gene mutation

## Abstract

**Introduction:**

We present a case of autosomal dominant Charcot–Marie–Tooth disease type 4B3 (CMT4B3) in a family caused by a novel *SBF1* missense mutation.

**Methods:**

Two patients, a mother and daughter, were recruited from our hospital. Both exhibited early-onset symptoms, including distal muscle atrophy of the limbs, without cranial nerve involvement. Electromyography was performed to assess nerve amplitudes and conduction velocities. Whole-exome sequencing (WES) and Sanger sequencing were performed to identify genetic mutations.

**Results:**

Electromyography revealed a significant decline in nerve amplitudes, while the nerve conduction velocities (NCVs) remained normal in the extremities. Sequencing identified a novel missense mutation (c.1398C > A, p.H466Q) in exon 13 of the *SET binding factor 1* (*SBF1*) gene in both patients, indicating an autosomal dominant inheritance pattern.

**Discussion:**

Pathogenicity and protein predictions suggest that the myotubularin-related protein 5 (MTMR5), encoded by the mutated *SBF1*, may possess an altered structure, resulting in disease. These findings will help expand the phenotypic and genetic spectrum of CMT4B3.

## 1 Introduction

Charcot–Marie–Tooth disease (CMT), also known as hereditary motor and sensory neuropathy (HMSN), is a rare, genetically heterogeneous group of disorders characterized by peripheral neuropathies. These disorders involve progressive atrophy of the muscles in the extremities and a decline in motor and sensory functions (1, 2). Based on the differences in nerve conduction velocities (NCVs) detected by electrophysiological studies, CMT is classified into three types: demyelinating (CMT1), axonal (CMT2), and intermediate (DI-CMT) (3, 4). Autosomal recessive forms of CMT1, referred to as CMT4 (AR-CMT1), include a group of subtypes known as CMT4B, which are associated with genes of the myotubularin-related protein (MTMR) family, namely, CMT4B1 (MTMR2, OMIM*603557) (5), CMT4B2 (MTMR13*/SBF2*, OMIM*607697) (6), and CMT4B3 (MTMR5*/SBF1*, OMIM*603560) (7). These genes are primarily involved in regulating endo-lysosomal trafficking (8). Mutations in these genes can lead to focal myelin out-folding and secondary axonal loss in peripheral nerves (4). To date, only seven families with *SBF1* mutations have been described (7, 9–16), all of which have autosomal recessive inheritance. In this study, a novel missense mutation in the *SBF1* gene was identified in a heterozygote mother and daughter with complex peripheral neuropathy, demonstrating that this mutation could alter the inheritance pattern and induce autosomal dominant inheritance of CMT4B3.

## 2 Methods

**Figure 1:**
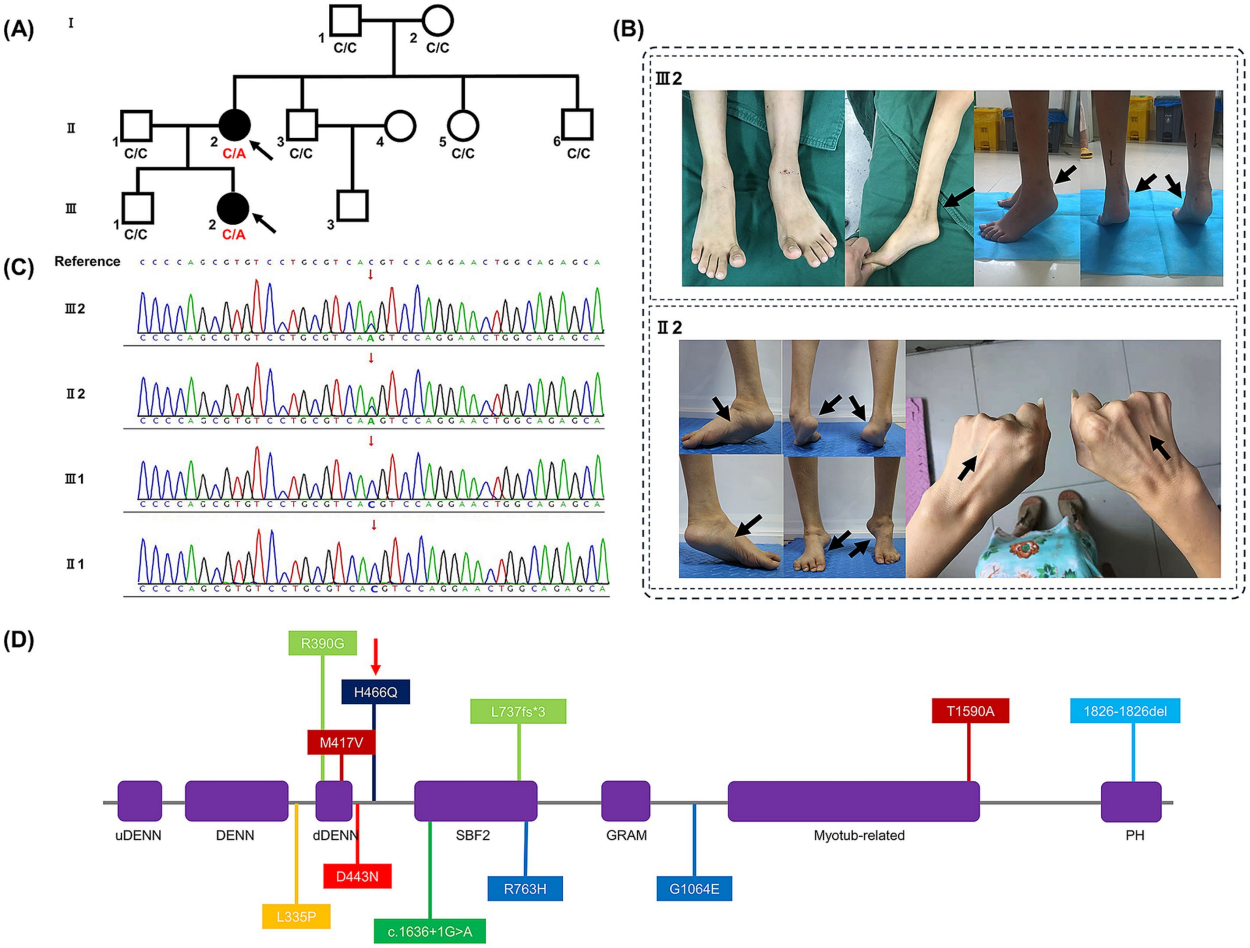
**(A)** Family pedigree demonstrating the mutated *SBF1* gene was inherited dominantly from patient II2 to III2 (affected members are shown in black, and the probands are indicated by black arrows). **(B)** Clinical phenotypes of patients II2 and III2. After first surgery of patient III2, her Achilles tendon contracture progressively still rendered both heels of her feet difficult to land, as indicated by black arrows. Symmetric atrophy of the lower limbs with high arches deformity and clubfeet, and symmetric atrophy of the upper limbs of patient II2, are also indicated by black arrows. **(C)** Results of Sanger sequencing in family members II1, II2, III1, and III2. **(D)** Scheme of the MTMR5 protein secondary structure. Abbreviations: uDENN, upstream DENN; DENN, differentially expressed in normal and neoplastic cells; dDENN, downstream DENN; GRAM, glucosyltransferases, Rab-like GTPase activators and Myotubularins; PH, Pleckstrin homology. Colors: Purple, domains; deep red, Korean family; red, Saudi family; orange, Syrian family; light green, Spanish family; green, Bedouin family; light blue, British family; blue, Italian family; deep blue, our variant (indicated by a red narrow).

This study was performed in accordance with the principles of the Declaration of Helsinki. Approval was granted by the Medical Ethics Committee of Zhongnan Hospital of Wuhan University (No. 2023296 K). Two patients (Patient II2 and III2) with a mother-daughter relationship (Figure 1A) were first seen at Zhongnan Hospital of Wuhan University in July 2019. Written informed consent was obtained from all the family members for all procedures. Senior doctors specializing in CMT diseases performed clinical assessments, including medical histories and neurological examinations. Electromyography was used to assess motor and sensory conduction velocities of various nerves in the upper and lower limbs in both patients. Brain and spinal MRIs were performed to investigate potential central nervous system impairment in patient III2. Patient III2 was admitted to the hospital for the treatment of bilateral Achilles tendon contractures. During the Achilles tendon release procedure, a small piece of the gastrocnemius muscle (approximately 8 mm^3^) was excised for further biopsy. The procedure was carried out by two senior orthopedic surgeons with over 10 years of surgical experience. Muscle biopsies were analyzed using hematoxylin and eosin (H&E), ATPase, NADH-TR, and modified Gomori trichrome staining techniques. Genetic analysis, including pathogenicity and protein prediction, was conducted by collecting blood samples from all family members to identify underlying genetic causes. Details of the Whole-exome sequencing (WES) (conducted by We-Health Biomed. Tech. Co., LTD), Sanger sequencing (conducted by Berry Genomics Co., LTD), and protein analysis are demonstrated in Supplementary material 1.

## 3 Results

### 3.1 Clinical features

#### 3.1.1 Patient II2

Here, we present the case of a 33-year-old female patient with an uneventful birth and normal early development. Her parents’ marriage was non-consanguineous. An abnormal gait was first detected at the age of 5 years, which progressively led to muscle weakness in the distal lower limbs and clubfoot. She could walk without any assistance for a maximum of 20 min. Physical examination revealed muscle atrophy above the elbow and knee (Figure 1B), proximal muscle weakness above the elbow and knee flexion and extension, decreased tendon reflex, mild sensory deficit, and additional symptoms including high arches, stiff gait, Gower’s maneuver, clubfeet, and gastroparesis.

#### 3.1.2 Patient III2

The patient was first examined in our consulting room around the age of 6 years. She had experienced an unsteady gait and heel lift since the age of 2. At the age of 5, she underwent surgery to externally release her Achilles tendon. Seven months after surgery, difficulty with heel contact to the ground gradually emerged (Figure 1B). We conducted several physical examinations and found that while her activities of daily living and sensory perception were normal, she exhibited other symptoms, including weakness in distal muscles (< 4 levels), muscle atrophy below the elbow and knee, high arches, Gower’s maneuver, clubfoot, and gastroparesis.

### 3.2 Electrophysiological findings

Electromyography demonstrated that the median and ulnar NCVs were > 38 m/s in both patients (Supplementary Table 1). The motor and sensory amplitudes of the upper and lower nerves showed a prominent decrease in both patients. No abnormalities were detected in the F-waves of the median and tibial nerves in either patient.

### 3.3 Imaging investigations

**Figure 2:**
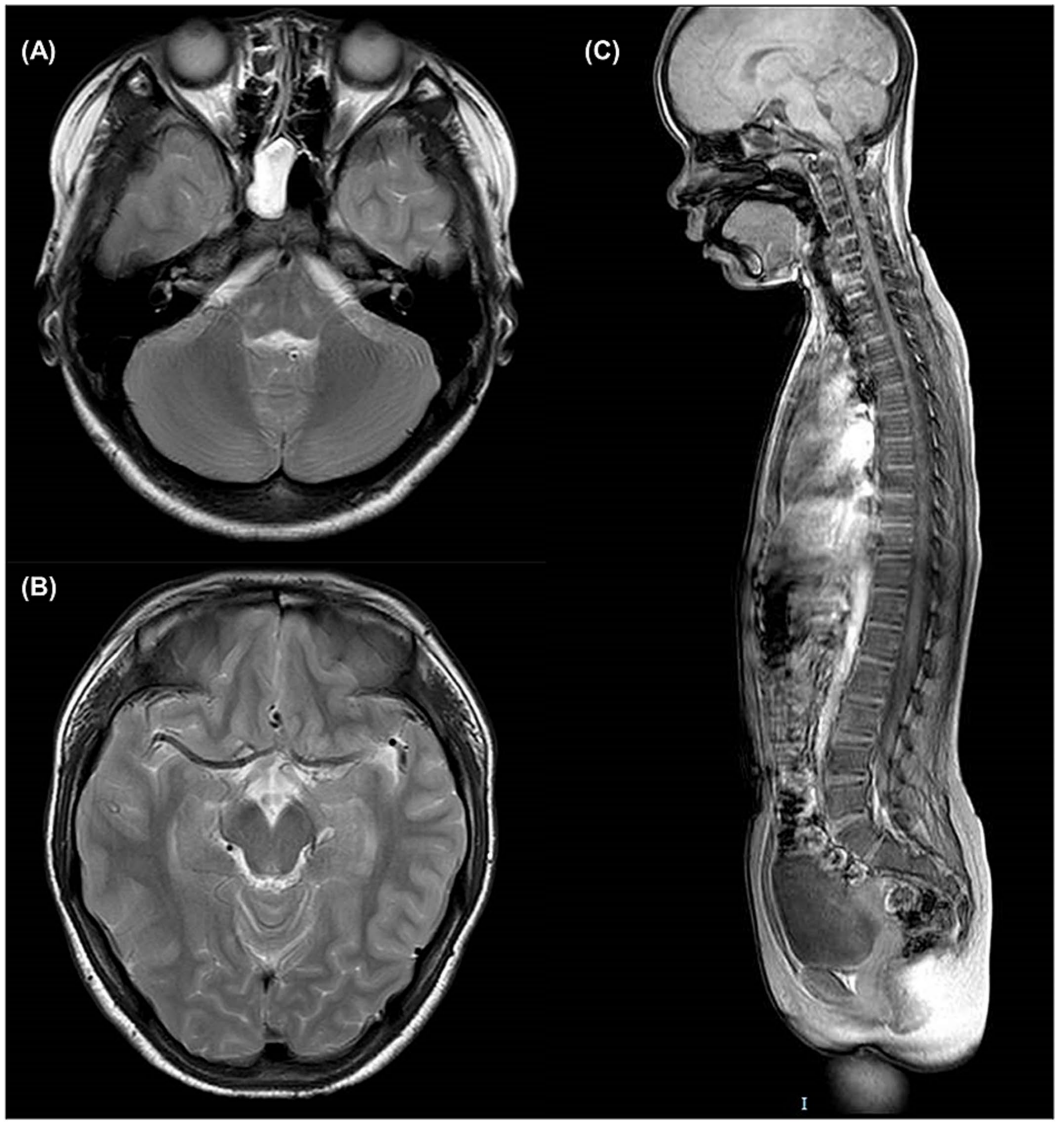
Brain and spinal MRI of patient III2. **(A)** T2-weighted axial slice at the level of pons. **(B)** T2-weighted axial slice at the level of mesencephalon. **(C)** T1-weighted sagittal slice of the entire spine.

No obvious lesions were observed in the brain and spinal MRI of patient III2 (Figure 2), particularly at the level of the mesencephalon and pons, where the “fork and bracket” signs were previously reported in Syrian (16) and Bedouin (9) families.

### 3.4 Histological study

**Figure 3:**
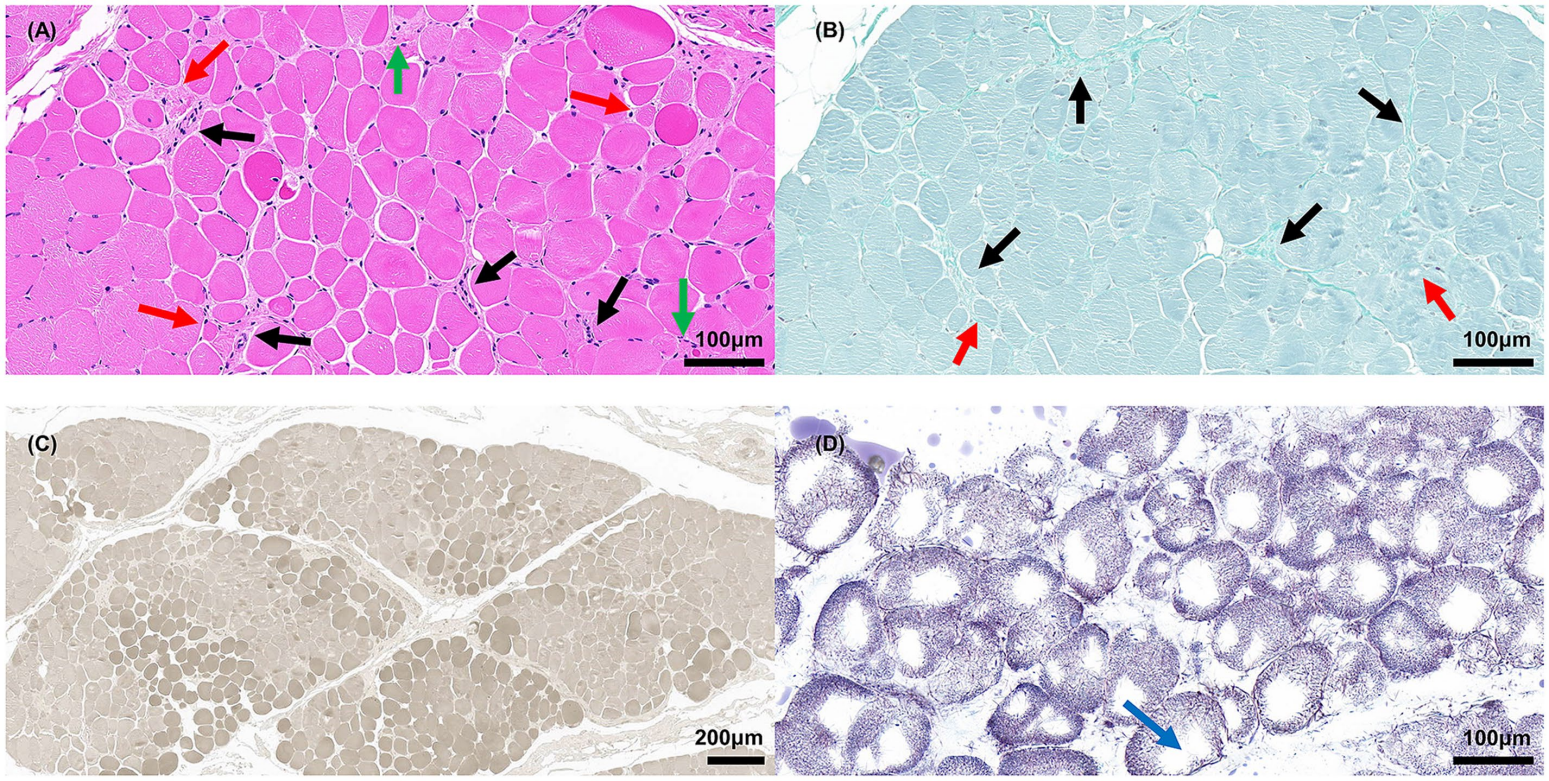
Muscle biopsies of gastrocnemius from patient III2. **(A)** H&E staining; red arrow indicates atrophic myofibers, black arrow indicates fibrosis, green arrow indicates aggregative myonuclei, scale bar = 100 μm. **(B)** Modified Gomori trichrome staining; the red arrow indicates atrophic myofibers; the black arrow indicates fibrosis, scale bar = 100 μm. **(C)** ATPase staining (pH 9.8), scale bar = 200 μm. **(D)** NADH-TR staining; blue arrow indicates vacuoles, scale bar = 100 μm.

Muscle biopsies of the gastrocnemius from patient III2 (Figure 3) revealed small groups of muscle fiber atrophy (red arrows) and fibrosis (black arrows), with variations in myofiber diameter and fiber splitting with cavity formation observed in the H&E and modified Gomori trichrome staining analyses. An increase in the number and aggregation of myonuclear cells (green arrows) was also noted. The color depth variation of muscle fibers in ATPase staining reflected the differentiation between slow- and fast-twitch fibers. Both slow and fast myofiber atrophies indicated the presence of neurogenic lesions. NADH-TR staining showed dispersed muscle fibers, with one or several large vacuoles (blue arrow) appearing in the middle, indicating muscle fiber damage.

### 3.5 Genetic analysis and pathogenicity prediction

After conducting tests and analysis, the mean total sequencing yield of WES samples was approximately 14.63 gigabases per sample, with a 99.62% coverage rate for the targeted exon regions (≥10×). Compared to other family members without relevant symptoms, only 3 mutated genes have been identified in patient II2 and III2 in WES test, which were *SCN10A*, *PRDM12*, *SBF1*, respectively. Given to the clinical features and electromyographic study, mutated *SBF1* has been considered the main pathogenesis incurring to CMT4B3, and the remaining two mutations might be synonymous or non-pathogenic. Patient III2 and II2 were found to have a novel *SBF1* non-synonymous missense mutation (c.1398 Cytosine to Adenine [c.1398C > A] in exon 13 of mRNA) on one allele (chr22:50903281). This mutation resulted in a change at the 466th amino acid of the protein, transitioning from histidine (His) to glutamine (Gln) (p.H466Q). Sanger sequencing also confirmed the mutation at this site (Figure 1C), which has not been previously reported in the literature or listed in dbSNP yet. This mutation was not indexed in the Genome Aggregation Database in East Asian race (gnomAD-EAS), Exome Aggregation Consortium (ExAC), or 1,000 Genomes Project (1000G) databases. Base on the guideline of American College of Medical Genetics and Genomics (ACMG) (17), this mutation has met the pathogenic criteria: 1 moderate (**PM2**) and 2 supporting (**PP2, PP3**), which should be classified as a Variant of Uncertain Significance (VUS). According to the scores of *in silico* predictive analysis tools: FATHMM **[−2.26]**, PolyPhen-2 **[0.994]**, MutationTaster **[1]**, PROVEAN **[−7.14],** and SIFT **[0.002]**, this mutation was predicted to be detrimental. Given the clinical features and electromyographic findings, the mutated *SBF1* gene was considered to be the primary pathogenic factor of CMT4B3. And according to Mendel’s laws of inheritance, this type of mutation should be a dominant mutation.

### 3.6 Protein analysis

**Figure 4:**
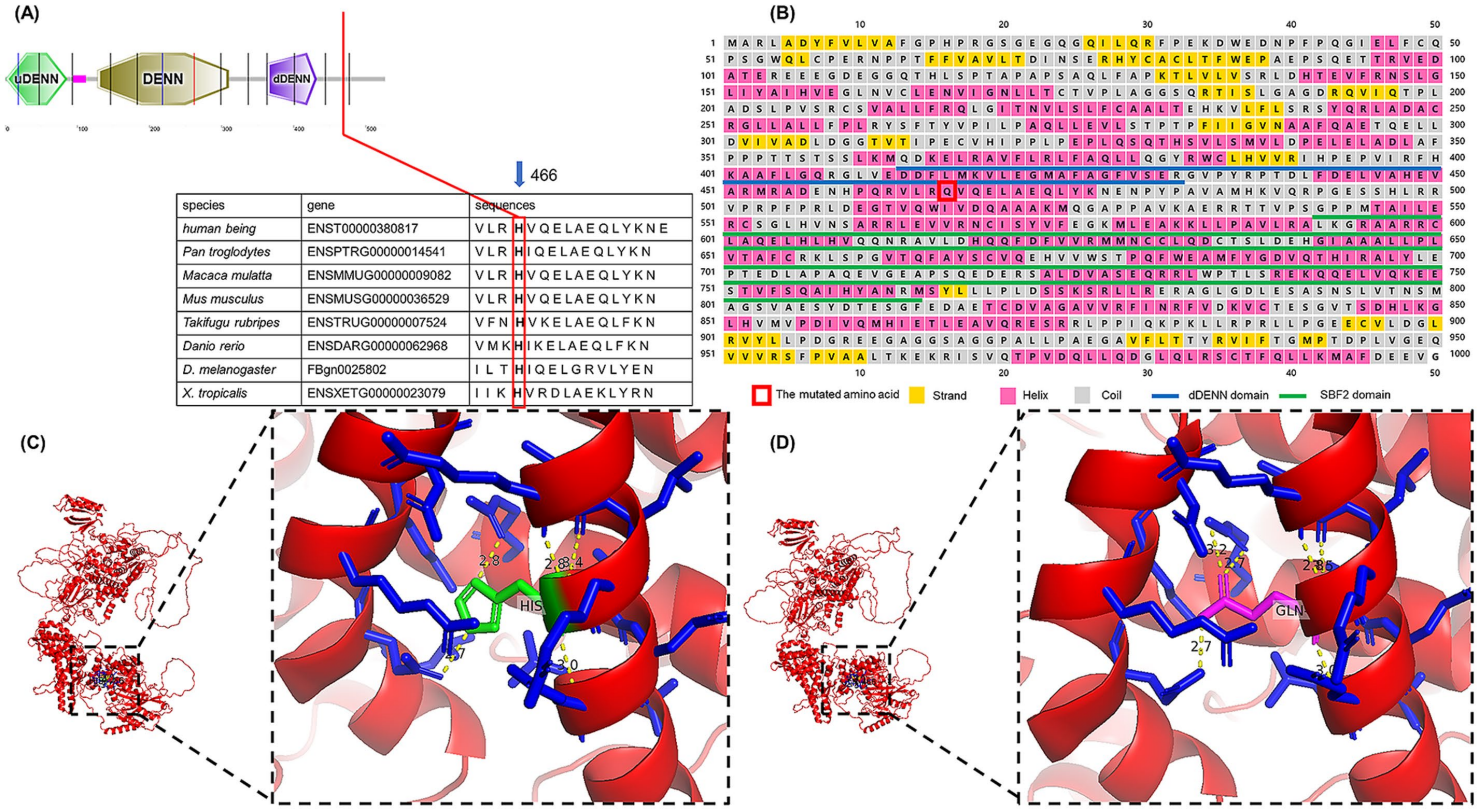
**(A)** Alignment of amino acid sequences across species. **(B)** The first 1,000 amino acids were extracted to predict the secondary structure and domain. **(C)** Wild-type MTMR5 protein; red indicates the protein body, blue indicates residues around the 466th amino acid, green indicates the 466th amino acid, namely His; yellow indicates hydrogen bonds. **(D)** Mutant MTMR5 protein; the red color indicates the protein body, the blue color indicates the residues around the 466th amino acid, the magenta color indicates the 466th amino acid, namely Gln; the yellow color indicates the hydrogen bonds.

We found that the *SBF1* gene was highly conserved at the mutation site in all tested species (Figure 4A). The PhastCons score (1.000) and the phyloP score (4.449) also indicated high conservation. According to the results of PSIPRED 4.0, the 466^th^ mutated amino acid in MTMR5 formed an alpha helix and was located between the dDENN and SBF2 domains (Figure 4B), similar to the wild-type MTMR5 (data source: www.uniprot.org/uniprotkb/O95248/feature-viewer). The three-dimensional structures of the proteins are shown in Figures 4C,D. Amino acid residue 466th may be structurally interconnected with the surrounding amino acid residues (residues nos. 370–377, 454–446, 463–471), whereas the *α*-helices formed by the latter two sets of residues form a triangular pocket structure with the α-helix formed by the first set residues of dDENN domain, which are located on the outer side of the MTMR5 protein, and may co-exercise dDENN domain function.

## 4 Discussion

Here, we describe two patients, a mother and daughter, who suffered from dominant CMT4B3 caused by a novel *SBF1* missense mutation. Both patients exhibited muscle atrophy and weakness in the extremities, particularly in the lower limbs, as confirmed by muscle biopsies and electromyography. The mutation site c.1398C > A (p.H466Q) in the *SBF1* gene, present on one allele in heterozygotes, was identified using WES and Sanger sequencing. This mutation was predicted to be detrimental, likely affecting the protein’s structure.

Several families with *SBF1* variants have been reported to exhibit significant clinical manifestations. Specific information about the mutation is provided in Supplementary Table 2. Both patients in this study were found to have autosomal recessive inheritance and heterogeneous clinical manifestations. Here, we summarize the sites of mutant amino acids in seven previously reported cases along with our variant, as shown in Figure 1D.

In our study, the clinical features of atrophy and weakness in the upper and lower limbs in two patients corresponded to those of CMT4 reported by Nakhro et al. in Korean family (7). However, their electrophysiological findings were distinct. NCVs >38 m/s in the median and ulnar nerves, along with markedly decreased amplitudes in both patients, more closely resembled the phenotype of the axonal form (CMT2) (18, 19). This is consistent with the CMT4B3 cases in other families, except for the Korean family. In addition, cranial nerve neuropathies and syndactyly observed in several familial cases are not consistent with the peripheral neuropathy due to demyelination seen in CMT4B3. Gene mutations in the Korean family may be predicted to be benign or tolerable, suggesting only a mild impact on protein function, whereas mutations in most reported cases were severe enough to cause polyneuropathy of the central nervous system. This large discrepancy in the genotype–phenotype correlation is likely due to specific *SBF1* mutations. Moreover, mutations affecting the dDENN motif or nearby amino acid residues have been identified in two families (p.D443N, p.L335P) with syndromic forms of axonal neuropathy (13–16). The novel mutation p.H466Q identified in our study is located near the dDENN motif, which may have contributed to the formation of a similar phenotype (20, 21).

It is unusual for the mutated *SBF1* gene to show a pattern of inheritance that deviates from the autosomal recessive manner observed in the seven cases reported before our research. The explanation for dominant inheritance is not clear currently. From a molecular perspective, MTMR5 functions as an adapter for the phosphatase MTMR2, forming a heterodimer through its coiled-coil domain (the C-terminal domain). This interaction regulates MTMR2’s catalytic activity on phosphatidylinositol 3-phosphate [PI (3)P] and influences its subcellular localization (22). It is possible that the mutant MTMR5 forms a heterodimer with MTMR2 that is difficult to depolymerize, producing a dominant negative effect leading to inhibition of the wild-type protein function (23). Additionally, MTMR5 may function as a guanine nucleotide exchange factor (GEF) mediated by the DENN domain, which activates Rab28 by promoting the exchange of GDP to GTP. This process converts inactive GDP-bound Rab proteins into their active GTP-bound forms (24), thereby regulating specific membrane trafficking and vesicular trafficking events (25, 26). The original DENN domain with more divergent domains encircling on its both sides, called uDENN and dDENN, may function together (27). DENNs form a conserved family of Rab GEFs. Based on the predicted protein structure, the mutated amino acid may impact Rab28 protein activation by changing the spatial structure around the DEMM domain, which in turn influences the transportation and conveyance of substances inside and outside the neuron cell (28). Exact pathogenesis requires further investigations in subsequent studies.

## 5 Conclusion

Here, we report a novel *SBF1* missense mutation that causes the autosomal dominant inheritance of CMT4B3 disease within a family. The mother and daughter exhibited neuropathy primarily in the extremities with muscle atrophy, but not in the cranial nerves. Genetic and protein analyses indicate that this *SBF1* missense mutation is the main cause of the disease; however, the mechanism underlying its action remains unknown. The identification of autosomal dominant inheritance will expand the genetic spectrum of CMT4B3.

## Data Availability

The datasets presented in this article are not readily available because of ethical and privacy restrictions. Requests to access the datasets should be directed to the corresponding authors.
