# Supplementary material for: A novel SBF1 missense mutation causes autosomal dominant Charcot–Marie–Tooth disease type 4B3
Source: Front Neurol. 2024 Nov 22;15:1495711. doi: 10.3389/fneur.2024.1495711 (PMC11633322; doi:10.3389/fneur.2024.1495711)
Supplement: Supplementary file 1 [file Data_Sheet_1.DOCX]

Supplementary Material

# Supplementary Data

## The process of WES test

WES was performed by collecting blood samples from all the family members to identify the underlying genetic causes. Genomic DNA was extracted from the peripheral blood samples by using blood DNA extraction kit (DP348-03, Tiangen, Shanghai, China) to construct a cDNA library. The total amount and quality of DNA were inspected by Onedrop OD1000 spectrophotometer and agarose gel electrophoresis. Exons were captured by using xGen Exome Research Panel v1.0 kit (Integrated DNA Technologies, Inc., Iowa, United States), and then were sequenced on the HiSeq 4000 sequencer (Illumina, San Diego, CA) to produce paired-end 150 bp reads sequence at a target depth of 100X. Original sequence alignment was performed by using Burrows-Wheeler Aligner (BWA) and SAMtools software. Duplicate data were removed by using Picard, and variant identifications were performed by using Genome Analysis Toolkit (GATK v3.70). Further annotation and filtering were done through Single Nucleotide Polymorphism Database (dbSNP; www.ncbi.nlm.nih.gov/snp/), Online Mendelian Inheritance in Man (OMIM; www.omim.org/) and ClinVar (www.ncbi.nlm.nih.gov/clinvar/). The 1000 Genomes Project (1000G; www.internationalgenome.org/), Exome Variant Server (EVS; evs.gs.washington.edu), Exome Aggregation Consortium (ExAC; exac.broadinstitute.org) and Genome Aggregation Database (gnomAD; gnomad.broadinstitute.org) were utilized to assess population variation frequency. Protein Variation Effect Analyzer (PROVEAN; provean.jcvi.org/index.php), Polyphen-2 (genetics.bwh.harvard.edu/pph2/) and Mutation Taster (www.mutationtaster.org) were used for pathogenicity analysis of mutated genes and proteins. Online tools PSIPRED 4.0 (bioinf.cs.ucl.ac.uk/psipred/) and Self-Optimized Prediction Method from Alignment (SOPMA; npsa-prabi.ibcp.fr/cgi-bin/npsa_automat.pl?page=npsa%20_sopma.html) were utilized to predict the secondary structure of the mutated protein.

## The process of Sanger Sequencing

Primers were synthesized based on the DNA fragments (hg19: chr22:50903261-50903301) to be sequenced. The forward primer sequence was 5’- GCCCTACGGACCTGTTCG-3’, and the reverse primer sequence was 5’- TGTACCTTGTGCATCGCCA-3’, with an annealing temperature of 60°C. Amplification was performed using the polymerase chain reaction (PCR) method. The PCR reaction system had a total volume of 25 µL, including 12.5 µL of mix, 1 µL of each primer at a concentration of 10 µmol/L, and 9.5 µL of ddH_2_O. The PCR cycling conditions were as follows: initial denaturation at 95°C for 10 minutes; followed by 35 cycles of 95°C for 30 seconds, 60°C for 30 seconds, and 72°C for 30 seconds; and a final extension at 72°C for 10 minutes. The PCR amplification products were sequenced using an ABI 3730xl sequencer (Applied Biosystems, USA) with Sanger sequencing. The sequencing results were analyzed by comparing them with the reference sequence using the 'Mutation Surveyor' software.

## The process of Protein analysis

Conservative analysis of amino acid sequence was performed firstly through online tool Clustal Omega for determining the conservation of protein primary structure. Orthologs amino acid sequences for SBF1 gene of several species including *human being, Pan troglodytes, Macaca mulatta, Mus musculus, Takifugu rubripes, Danio rerio, D. melanogaster and X. tropicalis* have been aligned through Clustal Omega ([www.ebi.ac.uk/Tools/msa/clustalo/](https://www.ebi.ac.uk/Tools/msa/clustalo/)). PhastCons and phyloP methods were also used to determine the grade of conservation of a given nucleotide. Secondly, online tools PSIPRED 4.0 (bioinf.cs.ucl.ac.uk/psipred/) and Self-Optimized Prediction Method from Alignment (SOPMA; npsa-prabi.ibcp.fr/cgi-bin/npsa_automat.pl?page=npsa%20_sopma.html) have been utilized to predict the secondary structure of the mutated protein. Pfam (pfam.xfam.org/) Databases have executed the forecast of domains of protein. Afterward, Swiss model (swissmodel.expasy.org/) and Pymol v2.6 have been used to execute the homologous modeling and bioinformatic prediction for the tertiary structure of protein, especially the intermolecular forces like hydrogen bonds.

# Supplementary Figures and Tables

## Supplementary Table

### Table 1 Electrophysiologic features of patient Ⅱ2 and Ⅲ2

|  | | Patients | | | |
| --- | --- | --- | --- | --- | --- |
|  | | Patient Ⅱ2 | | Patient Ⅲ2 | |
| Side | | Left | Right | Left | Right |
| Motor conduction | |  |  |  |  |
| Ulnar nerve | |  |  |  |  |
| Wrist - ADM | Lat, ms | 2.50 | - | 1.70 | 1.58 |
|  | Amp, mV | 3.90 ↓ | - | 5.60 ↓ | 6.50 |
|  | NCV, m/s | - | - | - | - |
|  | Dist, mm | - | - | - | - |
| Elbow - Wrist | Lat, ms | - | - | 4.33 | - |
|  | Amp, mV | - | - | 4.20 ↓ | - |
|  | NCV, m/s | - | - | 49.4 | - |
|  | Dist, mm | - | - | 130 | - |
| Upper arm - Elbow | Lat, ms | - | - | 5.58 | - |
|  | Amp, mV | - | - | 3.80 ↓ | - |
|  | NCV, m/s | - | - | 64.0 | - |
|  | Dist, mm | - | - | 80.0 | - |
| Median nerve | |  |  |  |  |
| Wrist - APB | Lat, ms | 2.33 | - | 2.14 | 2.17 |
|  | Amp, mV | 8.20 | - | 9.10 | 9.90 |
|  | NCV, m/s | - | - | - | - |
|  | Dist, mm | - | - | - | - |
|  | F wave, ms | 21.1 | - | 35.1 | 22.7 |
| Elbow - Wrist | Lat, ms | - | - | 5.06 | - |
|  | Amp, mV | - | - | 7.10 | - |
|  | NCV, m/s | - | - | 53.1 | - |
|  | Dist, mm | - | - | 155 | - |
| Tibial nerve | |  |  |  |  |
| Ankle - AH | Lat, ms | 2.82 | - | 3.04 | 2.50 |
|  | Amp, mV | 2.60 ↓ | - | 8.20 | 7.10 |
|  | NCV, m/s | - | - | - | - |
|  | Dist, mm | - | - | - | - |
|  | F wave, ms | 22.9 | - | 32.3 | 30.7 |
| Common peroneal nerve | |  |  |  |  |
| Ankle - EDB | Lat, ms | 3.40 | - | 2.21 | 2.52 |
|  | Amp, mV | 2.10 ↓ | - | 1.32 ↓ | 1.88 ↓ |
|  | NCV, m/s | - | - | - | - |
|  | Dist, mm | - | - | - | - |
| Fibular head - Ankle | Lat, ms | 8.83 | - | 7.00 | 6.88 |
|  | Amp, mV | 1.98 ↓ | - | 1.37 ↓ | 2.30 ↓ |
|  | NCV, m/s | 47.9 | - | 41.8 | 41.3 |
|  | Dist, mm | 260 | - | 200 | 180 |
| Sensory conduction | |  |  |  |  |
| Ulnar nerve | |  |  |  |  |
| Wrist - Finger Ⅴ | Lat, ms | 1.42 | 1.44 | 1.09 | 1.22 |
|  | Amp, μV | 17.0 ↓ | 3.00 ↓ | 32.4 | 33.6 |
|  | NCV, m/s | 56.3 | 55.6 | 59.6 | 57.4 |
|  | Dist, mm | 80.0 | 80.0 | 65.0 | 70.0 |
| Wrist - Finger Ⅳ | Lat, ms | - | 1.48 | - | - |
|  | Amp, μV | - | 2.30 ↓ | - | - |
|  | NCV, m/s | - | 54.1 | - | - |
|  | Dist, mm | - | 80.0 | - | - |
| Median nerve | |  |  |  |  |
| Wrist - Finger Ⅱ | Lat, ms | 1.35 | - | 1.38 | 1.26 |
|  | Amp, μV | 37.7 | - | 42.7 | 48.9 |
|  | NCV, m/s | 59.3 | - | 58.0 | 63.5 |
|  | Dist, mm | 80.0 | - | 80.0 | 80.0 |
| Superficial peroneal nerve | |  |  |  |  |
| Stimulation - Ankle | Lat, ms | - | - | 1.49 | 1.34 |
|  | Amp, μV | - | - | 13.1 | 18.8 |
|  | NCV, m/s | - | - | 50.3 | 56.0 |
|  | Dist, mm | - | - | 75.0 | 75.0 |
| Lower leg - Ankle | Lat, ms | 1.72 | - | 1.54 | 1.34 |
|  | Amp, μV | 23.5 | - | 11.1 | 17.6 |
|  | NCV, m/s | 49.4 | - | 48.7 | 56.0 |
|  | Dist, mm | 85.0 | - | 75.0 | 75.0 |
| Sural nerve | |  |  |  |  |
| Mid-Calf - Lateral Malleolus | Lat, ms | 1.65 | - | 1.60 | 1.42 |
|  | Amp, μV | 14.5 | - | 5.20 ↓ | 2.50 ↓ |
|  | NCV, m/s | 54.5 | - | 50.0 | 52.8 |
|  | Dist, mm | 90.00 | - | 80.0 | 75.0 |
| Mid 2 - Lateral Malleolus | Lat, ms | 1.67 | - | - | - |
|  | Amp, μV | 12.1 | - | - | - |
|  | NCV, m/s | 53.9 | - | - | - |
|  | Dist, mm | 90.0 | - | - | - |

Abbreviation: ADM=Abductor Digiti Minimi; APB=Abductor Pollicis Brevis; AH=Ankle Hallucis; EDB=Extensor Digitorum Brevis; Mid 2=Middle lower third of calf; Lat=Latency; Amp=Amplitude; NCV=Nerve Conduction Velocity; Dist=Distance between pole and measuring point; - =undetected.

### Table 2 Seven cases having been reported until now

| Case ID | Reporting year | Located region | Mutation site | Mode of inheritance | Main clinical manifestations | Neuropathy | Reference |
| --- | --- | --- | --- | --- | --- | --- | --- |
|  | 2013 | Korean | c.1249A>G (p.M417V); c.4768A>G (p.T1590A) | AR | atrophy and weakness of limbs; difficulty walking | Demyelinating | (1) |
|  | 2014 | Saudi | c.1327G>A (p.D443N) | AR | microcephaly; strabismus; syndactyly | Axonal | (2, 3) |
|  | 2016 | Syrian | p. L335P | AR | “fork and bracket” syndrome; microcephaly; cranial nerve neuropathies; intellectual disability | Axonal | (4, 5) |
|  | 2017 | Spanish | c.1168C>G (p.R390G); c.2209-2210del (p. L737fs*3) | AR | axonal neuropathy; cranial nerve involvement; additional neurological and skeletal features | Axonal | (6) |
|  | 2018 | Bedouin | c.1636+1G>A splice-site mutation | AR | polyneuropathy; muscle weakness of extremities; positive pyramidal signs; multiple cranial neuropathies | Axonal | (7) |
|  | 2020 | British | c.5477-5478del (p.1826-1826del) | AR | syndactyly; pes cavus; muscle weakness; vision problem; swallowing difficulties; imbalance | Axonal | (8) |
|  | 2021 | Italian | c.2291G>A (p.R763H); c.3194G>A (p.G1064E) | AR | motor and axonal polyneuropathy; talon-valgus-pronated clubfoot; Gowers’ sign; knee valgus | Axonal | (9) |

Abbreviation: AR=autosomal recessive.

**References**:

1. Nakhro K, Park JM, Hong YB, Park JH, Nam SH, Yoon BR, et al. SET binding factor 1 (SBF1) mutation causes Charcot-Marie-Tooth disease type 4B3. Neurology (2013) 81:165-73. doi: 10.1212/WNL.0b013e31829a3421

2. Alazami AM, Alzahrani F, Bohlega S, Alkuraya FS. SET binding factor 1 (SBF1) mutation causes Charcot-Marie-tooth disease type 4B3. Neurology (2014) 82:1665-6. doi: 10.1212/wnl.0000000000000331

3. Bohlega S, Alazami AM, Cupler E, Al-Hindi H, Ibrahim E, Alkuraya FS. A novel syndromic form of sensory-motor polyneuropathy is linked to chromosome 22q13.31-q13.33. Clin Genet (2011) 79:193-5. doi: 10.1111/j.1399-0004.2010.01524.x

4. Mégarbané A, Dorison N, Rodriguez D, Tamraz J. Multiple cranial nerve neuropathies, microcephaly, neurological degeneration, and "fork and bracket sign" in the MRI: a distinct syndrome. Am J Med Genet A (2010) 152a:2297-300. doi: 10.1002/ajmg.a.33417

5. Romani M, Mehawej C, Mazza T, Mégarbané A, Valente EM. "Fork and bracket" syndrome expands the spectrum of SBF1-related sensory motor polyneuropathies. Neurol Genet (2016) 2:e61. doi: 10.1212/nxg.0000000000000061

6. Manole A, Horga A, Gamez J, Raguer N, Salvado M, San Millán B, et al. SBF1 mutations associated with autosomal recessive axonal neuropathy with cranial nerve involvement. Neurogenetics (2017) 18:63-7. doi: 10.1007/s10048-016-0505-1

7. Flusser H, Halperin D, Kadir R, Shorer Z, Shelef I, Birk OS. Novel SBF1 splice-site null mutation broadens the clinical spectrum of Charcot-Marie-Tooth type 4B3 disease. Clin Genet (2018) 94:473-9. doi: 10.1111/cge.13419

8. Gang Q, Bettencourt C, Holton J, Lovejoy C, Chelban V, Oconnor E, et al. A novel frameshift deletion in autosomal recessive SBF1-related syndromic neuropathy with necklace fibres. J Neurol (2020) 267:2705-12. doi: 10.1007/s00415-020-09827-y

9. Berti B, Longo G, Mari F, Doccini S, Piccolo I, Donati MA, et al. Bi-allelic variants in MTMR5/SBF1 cause Charcot-Marie-Tooth type 4B3 featuring mitochondrial dysfunction. BMC Med Genomics (2021) 14:157. doi: 10.1186/s12920-021-01001-1
